# Introgressed *Brassica napus* × *Sinapis alba* enhance resistance to the crucifer flea beetle (*Phyllotreta cruciferae*)

**DOI:** 10.1002/ps.71097

**Published:** 2026-07-14

**Authors:** Aziz Ullah, Altaf Hussain, Vicky Roslinsky, Sally L. Vail, Boyd A. Mori

**Affiliations:** ^1^ Department of Agricultural, Food & Nutritional Science University of Alberta Edmonton AB Canada; ^2^ Saskatoon Research and Development Centre, Agriculture and Agri‐Food Canada Saskatoon SK Canada

**Keywords:** Glucosinolate profiles, host plant resistance, intergeneric irogression, *Phyllotreta cruciferae*

## Abstract

**BACKGROUND:**

The crucifer flea beetle (*Phyllotreta cruciferae*) is an invasive pest of Canadian canola, and its management would benefit from durable host plant resistance. *Brassica napus* has limited inherent insect resistance, whereas *Sinapis alba* exhibits resistance or tolerance to multiple insect pests. Consequently, introgressed lines combining *B. napus* and *S. alba* offer opportunities to identify resistant traits and develop canola‐quality germplasm with enhanced defenses. This study examined host plant resistance *B. napus* lines carrying *S. alba* introgressions and explored underlying resistance mechanisms. Intergeneric hybrids were generated via embryo rescue, followed by multiple backcrosses to *B. napus* and the development of double haploid mapping populations. Seven intergeneric hybrids and their parental lines were used in feeding bioassays conducted with *P. cruciferae* collected on the Canadian Prairies. Glucosinolate profiles, chlorophyll content, trichome density and linear electron flow (LEF) were quantified in seedlings of introgressed and parental genotypes.

**RESULTS:**

Fifteen distinct glucosinolate profiles were identified, with significant variation among lines. Introgressed lines with elevated glucosinolate concentrations sustained significantly less feeding damage than those with lower levels. This resistance was linked to chromosome SA02, which harbors several glucosinolate quantitative trait loci. Higher glucosinolate levels were positively correlated with chlorophyll content and LEF. Trichomes were present only in the *S. alba* parental line, indicating that constitutive physical defenses played a minor role compared to chemical defenses.

**CONCLUSION:**

These findings underscore the potential of introgressed lines to enhance defense mechanisms against flea beetle damage and support research to optimize host plant resistance in canola breeding programs. © 2026 His Majesty the King in Right of Canada and The Author(s). *Pest Management Science* published by John Wiley & Sons Ltd on behalf of Society of Chemical Industry. Reproduced with the permission of the Minister of Agriculture and Agri‐Food.

## Graphical abstract

This study demonstrates that *Brassica napus* lines with *Sinapis alba* introgressions enhanced resistance against *Phyllotreta cruciferae*, resulting in reduced feeding damage and improved seedling physiological performance. The introgressed lines showed strong potential as genetic resources for developing more resilient and sustainable canola varieties.

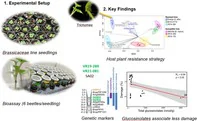

## 1 INTRODUCTION

Grown for edible oil, livestock feed and biofuel feedstocks, canola (*Brassica napus* L. and *Brassica rapa* L.) and oilseed rape (*Brassica napus* L.) are one of the most important global oilseed crops.^1^ Canada is the leading world producer and exporter of canola,^2^ with production exceeding 8.6 million ha annually^3^ and contributing over CA$44 billion to the national economy.^4^ Canola production is constrained by both abiotic stresses, including extreme temperature, excessive moisture, and drought,^5^ and biotic stresses such as insects and disease.^6^, ^7^ On the Canadian Prairies, striped (*Phyllotreta striolata* F.) and crucifer (*Phyllotreta cruciferae* Goeze) flea beetles (Coleoptera: Chrysomelidae) are the most significant insect pests of canola,^8^ causing millions of dollars in losses each year.^9^, ^10^, ^11^ To sustain canola production under increasing environmental and regulatory pressures, next‐generation, integrated pest management (IPM) strategies will be required that provide durable early‐season protection and improve the resilience of management programs.

Management of flea beetles in canola relies on a combination of cultural practices and chemical control; however, current production systems in Canada remain heavily dependent on insecticide seed treatments.^8^, ^11^, ^12^, ^13^ Insecticide seed treatments (e.g. neonicotinoids, diamides) provide early‐season protection during crop establishment and can reduce the need for foliar insecticide applications.^12^, ^14^ However, their long‐term sustainability is increasingly questioned. The Pest Management Regulatory Agency (PMRA) previously re‐evaluated neonicotinoids owing to concerns regarding nontarget effects, and although their continued use in canola seed treatments has been permitted with mitigation measures,^15^, ^16^ regulatory scrutiny remains ongoing. Additionally, *P. striolata* has been shown to be more tolerant to neonicotinoid seed treatments than *P. cruciferae*,^17^ raising concerns about reduced efficacy and the potential for resistance development under continued reliance on these chemistries. Together, these factors highlight the need for alternative and complementary strategies within IPM frameworks. Canola seedlings are especially vulnerable to flea beetle injury, as adults rapidly colonize and feed extensively on cotyledons, causing characteristic shot‐hole damage that reduces photosynthetic capacity, delays development, and can result in stand and yield losses.^18^, ^19^, ^20^ Although insecticide‐based tools remain essential components of flea beetle IPM, their performance can vary with environmental conditions, pest pressure and flea beetle species.^17^ Thus, it is crucial to develop alternative and complementary management strategies, including host plant resistance, within IPM programs to enhance crop resilience.

Host plant resistance is recognized as an integral component of IPM as it is easy to use, generally inexpensive to the end user, and compatible with other tactics.^21^ Although some resistance to flea beetles has been documented across the Brassicacaeae, commercial canola varieties have historically shown little consistent resistance to *Phyllotreta* spp. or other insect pests.^22^, ^23^, ^24^, ^25^, ^26^

By contrast, white (or yellow) mustard (*Sinapis alba* L.) has demonstrated resistance or tolerance against *P. cruciferae*,^23^ making it a valuable genetic resource for developing resistant canola quality lines. Resistance in *S. alba* has been attributed to a combination of antixenosis and tolerance, with glucosinolate composition [particularly hydroxybenzyl glucosinolate (sinalbin)] associated with reduced feeding, and rapid early growth contributing to tolerance of *P. cruciferae* injury.^27^, ^28^, ^29^ Glucosinolates are a class of sulfur‐ and nitrogen‐containing secondary metabolites characteristic of Brassicaceae, which play a key role in plant defense.^30^ Upon tissue damage, glucosinolates are hydrolyzed by the enzyme myrosinase to produce biologically active compounds such as isothiocyanates, nitriles and thiocyanates, many of which are toxic or deterrent to generalist herbivores. However, specialist herbivores such as flea beetles have evolved mechanisms to tolerate or even exploit glucosinolates as host‐recognition cues, complicating their role in resistance.^31^ Recent methodological advancements and the development of introgressed germplasm provide opportunities to better resolve these mechanisms and identify traits for resistance breeding.

Because commercial *S. alba* lacks the oil and meal quality required for commercial canola production, intergeneric introgression between *S. alba* and *B. napus* (hereafter, introgressed lines) has been pursued to transfer genomic regions potentially containing beneficial traits, including disease resistance, insect resistance and abiotic stress tolerance, into canola‐quality *B. napus* backgrounds. However, the development of such introgressed lines is genetically challenging because of reproductive barriers, chromosome incompatibility, linkage drag, and the need to recover desirable agronomic and seed quality traits. Despite these challenges, previous studies have demonstrated the successful transfer of useful traits through interspecific and intergeneric hybridization in Brassica crops, including clubroot resistance and other stress‐related traits, aided by approaches such as embryo rescue, molecular markers and other biotechnological tools.^32^, ^33^, ^34^ Although introgressed lines have shown reduced flea beetle feeding (e.g. ^23^), resistance has been variable and the underlying mechanism remains poorly resolved, particularly ambiguity surrounding the role of glucosinolates, and the observation that other factors may be involved in resistance.^35^, ^36^ Recent methodological advancements and development of introgressed germplasm provides opportunities to resolve mechanisms and identify traits for resistance breeding.

In this study, we evaluated host plant resistance to *P. cruciferae* in introgressed *B. napus* × *S. alba* germplasm. *Phyllotreta cruciferae* continues to be the dominant flea beetle species across the Canadian Prairies^11^, ^20^ and poses the greatest early‐season damage risk to canola. Our objectives were to: (i) evaluate flea beetle feeding damage among introgressed lines, and (ii) identify potential physiological and biochemical traits associated with resistance or tolerance. Specifically, we examined relationships among flea beetle feeding and seedling glucosinolate profiles, chlorophyll content, linear electron flow (LEF) and biomass with the goal of identifying candidate resistance markers that support host plant resistance as a complementary, durable component of next‐generation IPM for canola.

## 2 MATERIALS AND METHODS

### 2.1 Development of intergeneric introgression lines

Intergeneric crosses were generated between *B. napus* (female; 46A65) and canola‐quality *S. alba* (male; SAB), which required ovule and embryo rescue techniques (explained in detail previously^37^), resulting in 0.35% intergeneric hybrids which were further confirmed using flow cytological evaluation and intermediate morphological characterization (Supporting information Fig. S1).^38^ The initial F_1_ intergeneric hybrids exhibited high pollen sterility (96–98%), as determined by acetocarmine staining during cytological observations of pollen mother cells. Therefore, the F_1_ hybrids were back‐crossed to *B. napus* (male; 46A65), and another round of embryo rescue^37^ was required (BC_1_F_1_), followed by a second back‐cross to *B. napus* (male; 46A65) to generate BC_2_F_1_ seed. The BC_2_F_1_ plants were subsequently self‐pollinated for four generations to produce the stable BC_2_F_4_ line BH14‐21772 (Table 1). Following this, a double haploid (DH) mapping population of ≈500 lines was generated between BH14‐21772 (female) and *B. napus* line YN01‐429 (male). In parallel plants from the BC_1_F_5_ and BC_2_F_3_ generations were screened for the presence of *S. alba* inserts and identified lines were backcrossed to the *B. napus* line N99‐508 to the BC_4_F_2_ stage (Fig. S1). Likewise, intergeneric hybridizations between mustard‐quality *S. alba* (female, Emergo) and *B. napus* (male, CrGC 5006) were generated through crossing followed by ovule and embryo rescue, then the F_1_ plant was back‐crossed to *B. napus* (Topas).^37^ Following that, BC_1_ plants were crossed then back‐crossed to *B. napus* (Westar) to generate BC_3_F_1_ plants from which DHs were generated (G. Séguin‐Swartz, personal communication). These DH lines also were screened for the presence of *S. alba* inserts and identified lines were again backcrossed to the *B. napus* line N99‐508 to the BC_3_F_2_ stage (Fig. S1).

**Table 1:** Breeding origin and total glucosinolate of introgression lines, parental *B. napus* and *S. alba* genotypes

Name	*S. alba* donor	Back‐cross (BC) *B. napus* parent	Description (Fig. S1)	Group	Total glucosinolates (nmol g^–1^ DW, mean ± SE)	Experiments
SAB			*S. alba* donor	*Donor*	Not measured^a^	1
Emergo			*S. alba* donor	*Donor*	3185 ± 190	1, 2
N99‐508			Back‐cross parent	Back‐cross parent	189 ± 7	1, 2
46A65			Back‐cross parent	Back‐cross parent	Not measured	1
BH14‐21772	SAB	46A65	*S. alba* SA03 insert, BC2	*S. alba* introgression lines	144 ± 6	1, 2
VR19‐176	SAB	N99‐508	*S. alba* SA05 insert; BC4	*S. alba* introgression lines	222 ± 7	1, 2
VR19‐208	SAB	N99‐508	*S. alba* SA07 insert; BC4	*S. alba* introgression lines	117 ± 6	1, 2
VR19‐289	SAB	N99‐508	*S. alba* SA02 insert; BC4	*S. alba* introgression lines	801 ± 25	1, 2
VR19‐133	SAB	N99‐508	*S. alba* SA01insert; BC4	*S. alba* introgression lines	Not measured	1
VR19‐156	SAB	N99‐508	*S. alba* SA03 insert; BC4	*S. alba* introgression lines	Not measured	1
VR21‐081	Emergo	N99‐508	*S. alba* SA02 insert; BC3	*S. alba* introgression lines	Not measured	1

^a^ Not measured, but canola‐quality *S. alba* (total glucosinolates <30 μmol g^–1^ seed).

Genomic DNA was isolated from young leaf tissue of 41 intergeneric lines (46A65 × SAB; 17 BC_1_F_5_, 24 BC_2_F_3_), 55 DH lines with Emergo as the *S. alba* source and 400 DH lines from the mapping population (BH14‐21772 × YN01‐429), using a sodium dodecyl sulfate extraction protocol.^39^ Putative *S. alba* introgression plants were screened with all mapped intron length polymorphism (ILP) markers (Fig. S2) as described previously.^40^ Two ILP markers from each unique alba insert were utilized to select suitable lines during all backcross generations (Fig. S1). In order improve overall coverage and to close gaps in the original map an additional 130 KASP markers were added utilizing a GBS approach as described in detail previously.^41^ The *S. alba* parental lines and a subset of the mapping population were analyzed and identified SNPs were converted to routine KASP markers (LGC Biosearch Technologies, Novato, CA, USA). For each KASP marker the reaction contained 50 ng genomic DNA, 4.0 μL KASP 2x Master Mix (LGC Biosearch Technologies) and 0.11 μL primer assay mix in a total volume of 8.0 μL. All amplifications were performed in a CFX96 Real‐Time Thermal Cycler (Bio‐Rad Laboratories, Hercules, CA, USA) using the touchdown PCR protocol as recommended by the manufacturer.

### 2.2 Insect bioassays

Seven different introgressed lines, along with their four *B. napus* and *S. alba* parental lines, were used in this study (Table 1). Seeds were sown in seven dram vials (26 mL) containing commercial potting mix (Sunshine Professional Growing Mix; SunGro Horticulture Canada, Seba Beach, AB, Canada) and grown for 6–8 days or until the cotyledons were fully developed in a controlled environmental chamber. The growth conditions for experiments 1 and 2 were maintained at 23 °C ± 3 during the day and 18 °C ± 3 at night, 55–60% relative humidity (RH), 16 h:8 h, light:dark photoperiod and 500 μmol m^–2^ s^–1^ light intensity.

For Experiment 1, spring‐ and summer‐generation crucifer flea beetles, *P. cruciferae*, were collected in June and August 2023, respectively, from canola on the Edmonton Research Station, University of Alberta, AB, Canada, (53.489814−113.537015°) using sweep nets. For Experiment 2, only spring *P. cruciferae* were collected in June 2024 from the same location using sweep nets. Collected beetles were reared on organic cabbage (*Brassica oleracea* L.) leaves inside 60 × 60 × 90‐cm mesh cages (Bugdorm, MegaView Science Co. Ltd, Taipei, Taiwan) maintained in a growth chamber under controlled conditions of 23 ± 3 °C, 55–60% RH and a 16 h:8 h, light:dark photoperiod. Beetles were used within 1 week of collection for bioassays.

### 2.3 Experiment 1 – feeding bioassays (11 lines)

No‐choice feeding bioassays were conducted to assess host plant resistance of a panel consisting of 11 lines (seven introgressed lines, four parental lines; Table 1). Experiments were conducted in parallel under controlled environmental conditions (*n* = 10 per line) with seven seedlings (of one line) placed into an experimental arena (960‐mL ventilated plastic container, Pint‐sized BugDorm; MegaView Science Co. Ltd) and 42 *P. cruciferae* added (six beetles per plant). Experimental arenas were arranged in a randomized orientation within the climate chamber to minimize positional effects. Seedlings were not physically measured before feeding assays to avoid damage at early developmental stages. Instead, seedlings were visually standardized to ensure similar cotyledon size and developmental stage across treatments, and any atypical or uneven seedlings were excluded. Proportion of cotyledon damage of each seedling was visually assessed after 72 h using a 0‐ to 10‐point scale which corresponded to the percentage of the cotyledon surface that was consumed by *P. cruciferae*.^42^, ^43^ Based on the scale, a rating of 0.5, 1.0 and 2.0 were assigned when 5%, 10% and 20% of the cotyledon surface was eaten.

### 2.4 Experiment 2 ‐ feeding bioassay (six lines)

A subset of six lines was selected from the initial panel of 11 Brassicaceae lines for further evaluation based on their feeding damage responses observed in Experiment 1 (Fig. 1). The experimental design, growth conditions, and feeding bioassay procedures used in this experiment were the same as those described for Experiment 1. This targeted selection allowed for a focused comparison among lines representing distinct resistance phenotypes while maintaining adequate statistical power. The lines were sorted into three distinct groups from least susceptible to highly susceptible. The least susceptible group consisted of introgressed lines BH14‐21772 and VR19‐289 because these lines had significantly lower feeding damage compared to all other lines (Fig. 1). The highly susceptible group consisted of introgressed lines VR19‐208 and VR19‐176 which had the highest levels of foliar damage (Fig. 1). Additionally, two parental lines, N99‐508 (*B. napus*) and Emergo (*S. alba*), were included for comparison. N99‐508 line served as the donor parent for all introgressed lines, whereas Emergo served as the donor parent for one introgressed line (VR21‐081). Because Emergo exhibited trichomes, it was included in this experiment to allow direct comparisons between parental and introgressed lines and to account for potential effects of trichome expression. The selection process resulted in a plant panel that enabled us to conduct meaningful statistical analyses of their resistance levels to *P. cruciferae* feeding. During this experiment, we also measured the following physiological and biochemical parameters from the selected six Brassicaceae lines.

**Figure 1:**
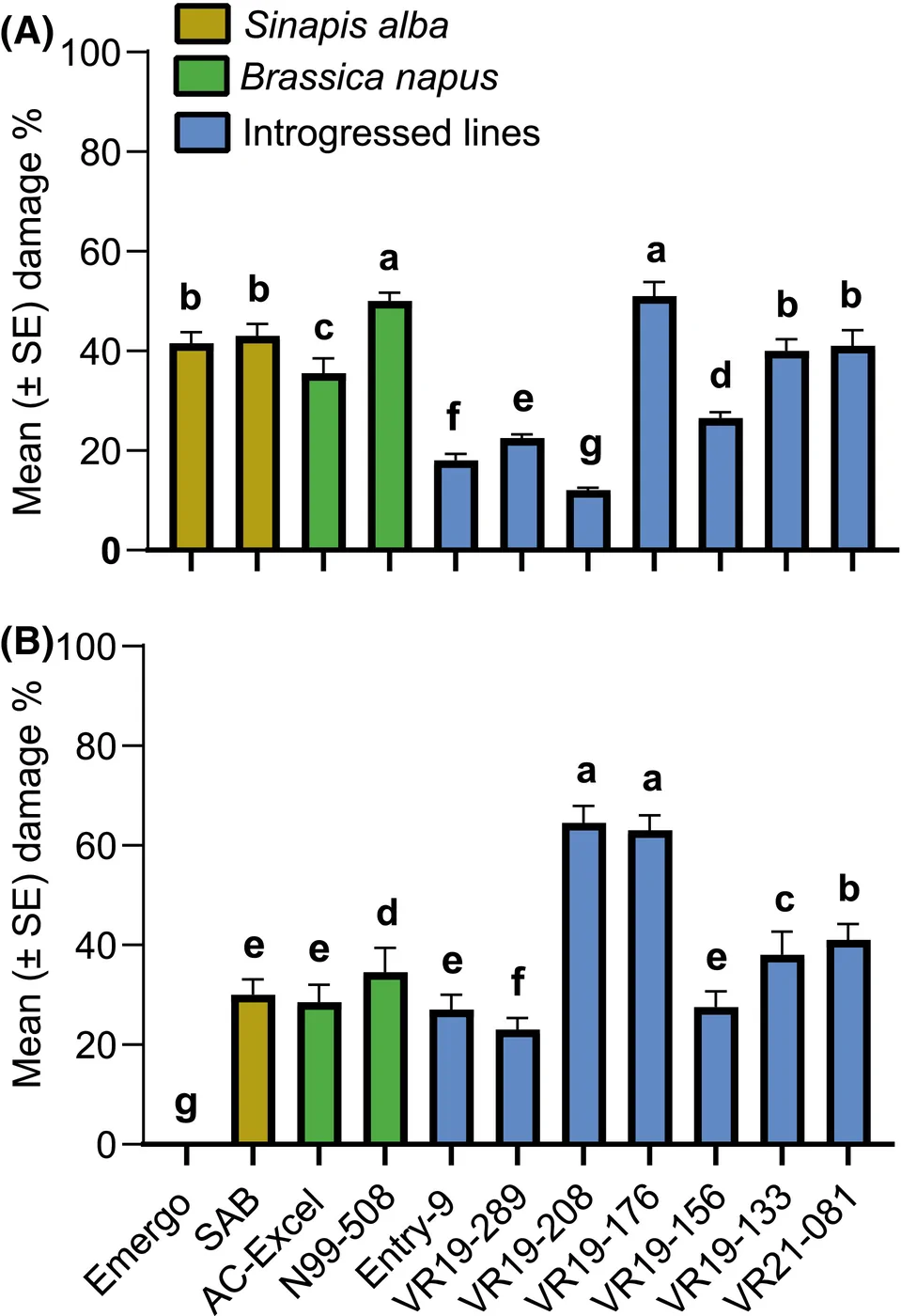
Comparison of feeding damage in the introgressed lines and controls (*B. napus* var. N99‐508, *S. alba* var. Emergo and SAB) for the (A) spring and (B) summer generations of *P. cruciferae* (*n* = 10).

### 2.5 Glucosinolate quantification

For glucosinolate analyses, a separate batch of seedlings from each line was grown concurrently under identical conditions as those used in the feeding bioassay (Experiment 2). These plants were not exposed to flea beetles. Cotyledon tissue was harvested and used for glucosinolate quantification on the same date as feeding bioassays began. Five experimental replicates per line were prepared. Each replicate consisted of pooled cotyledon tissue from seven seedlings (two cotyledons per seedling; 14 cotyledons total), corresponding to one experimental replicate (arena) used in the feeding bioassay. Glucosinolate content was quantified from cotyledons at the UVic‐Genome BC Proteomics Centre (University of Victoria, BC, Canada) using modified previous protocols.^24^, ^44^ Briefly, the cotyledons from each seedling were lyophilized at −50 °C for 72 h in a freeze‐drier (Labconco Corp., Kansas City, MO, USA). Following lyophilization, 20 mg tissue from each line were weighed and placed individually into 5‐mL safe‐lock Eppendorf tubes (*n* = 5 per line). Two metal beads, and 2 mL methanol, acetonitrile, dimethyl sulfoxide (DMSO) and dimethylformamide (DMF) (1:1:1:1 v/v) were added to each tube. Samples were homogenized using a mixer mill (MM 400; Retsch GmbH, Haan, Germany) at 30 Hz for 1 min, followed by cooling on ice for 2 min. The homogenization step was repeated three times. Samples were then centrifuged at 21000*g* and 10 °C for 10 min. Nine serially diluted calibration solutions containing 15 glucosinolate standards were prepared in the same extraction solvent, with concentrations ranging from 0.00001 to 20 μm. The 15 authentic standards were used to identify and quantify the concentrations of individual compounds: glucohirsutin (chemical purity: >98%), 4‐hydroxyglucobrassicin (>98%) (BioCrick, Sichuan, China), glucocapparin (>99%), glucoarabin (99%), glucoraphenin (99%), glucoalyssin (95%) (Biosynth, Berkshire, UK), epiprogoitrin (≥98%), glucoerucin (≥98%), gluconapin (≥98%), glucoiberin (≥98%), progoitrin (≥98%), glucoraphanin (≥98%), glucoraphanin (≥98%) (Extrasynthese, Genay, France), sinigrin (≥95%) and glucotropaeolin (≥98%) (Cayman Chem, Ann Arbor, MI, USA). An internal standard was not used because isotope‐labelled analogs were not available for the measured glucosinolates. Instead, external calibration (i.e. absolute peak area *versus* concentration) were used for quantitation, and to minimize analytical variation, all samples and calibration solutions were processed in parallel, and all glucosinolates detected in the samples were well within the linear ranges of liquid chromatography‐mass spectrometry (LC–MS) detection. For analysis, 20 μL each sample extract or calibration solution was mixed with 80 μL Optima™ LC/MS grade water (Fisher Scientific, Ottawa, ON, Canada). Aliquots (10 μL) of the resulting solutions were injected onto a C18 column (3.5 μm particle size; 3.0 × 100 mm; Agilent Technologies, Santa Clara, CA, USA) for analysis by ultra‐performance liquid chromatography‐multiple reaction monitoring‐mass spectrometry (UPLC‐MRM/MS) with (−) ion detection on a Acquity UPLC system (Waters, Milford, MA, USA) coupled to a QTRAP 6500 Plus mass spectrometer (Sciex, Framingham, MA, USA). Ammonium acetate buffer and methanol were used as the mobile phase for binary‐solvent gradient elution under optimized LC separation and MRM/MS detection conditions.

Glucosinolates were quantified using external calibration curves generated from authentic standards. Suitable stable isotope‐labelled glucosinolates, which are the preferred internal standards for LC–MS quantification, are not currently commercially available for the compounds analyzed; therefore, external (surrogate) standards with concentration–response calibration curves were used, as is standard practice for glucosinolate analysis.^45^, ^46^, ^47^ Calibration curves were generated using linear regression of peak areas across serially diluted standards, and sample concentrations were determined by interpolation. All samples and standards were processed and analyzed under identical conditions within the same analytical batches, and all analyte responses fell within the linear range of detection.

### 2.6 Chlorophyll content and linear electron flow

Chlorophyll content, typically measured in leaves or cotyledons, reflects the concentration of chlorophyll pigments within plant tissues and serves as an indicator of photosynthetic capacity. In plants, photosynthetic electron transport occurs primarily through LEF, a process that transfers electrons from water (H_2_O) to NADP^+^ generating nicotinamide adenine dinucleotide phosphate (NADPH) and adenosine triphosphate (ATP) required for the Calvin cycle (carbon fixation). Chlorophyll and LEF in cotyledons were measured nondestructively using a MultispecQ v2.0 handheld chlorophyll meter (PhotosynQ LLC, East Lansing, MI, USA) in conjunction with the photosynq Android application. The MultispecQ v2.0 device was connected to the application via Bluetooth using an Android smartphone.^48^ Measurements were collected from 10 biological replicate seedlings per line grown under the same experimental conditions described for Experiment 2.

### 2.7 Trichome density and length

Undamaged, healthy seedlings of all lines grown under the conditions described above were used for trichome quantification concurrently with the feeding bioassay. Trichome density was quantified by counting the number of trichomes within a randomly selected 1 cm^2^ area on both the adaxial, abaxial and hypocotyl surfaces of the cotyledons and the mean number were calculated for each seedling. Methods followed those described in Soroka *et al*.^49^ Fluorescence microscopy was conducted on an Axio Imager M_2_ microscope (Carl Zeiss Microscopy GmbH, Jena, Germany) equipped with a DAPI filter set (365 nm excitation and 445 nm emission) to detect Calcofluor White. Images were captured at 10× and 20× magnification using zen blue software (Zeiss). Trichome density and lengths of 10 randomly selected trichomes were measured with an optical graticule following^50^ and re‐measured by using imagej software (National Institute of Health, Bethesda, MD, USA).^51^ Trichome measurements were conducted using 10 biological replicate seedlings per line grown under the same experimental conditions described for Experiment 2.

### 2.8 Seedling biomass

The dry above‐ground biomass of undamaged 8‐day‐old seedlings from the six selected lines used in Experiment 2 was measured to assess baseline growth characteristics. Seedlings were grown under the conditions described above. Samples were lyophilized at −50 °C for 72 h in a freeze‐drier (Labconco Corp., Kansas City, MI, USA), after which dry biomass for each seedling was determined by using an analytical balance (AB204‐S; Mettler‐Toledo, Columbus, OH, USA). Biomass measurements were obtained from 10 biological replicate seedlings per line grown independently under the same conditions described for Experiment 2.

### 2.9 Statistical analyses

All statistical analyses were performed in R v4.1.0.^52^ *Phyllotreta cruciferae* feeding damage ratings from experiments 1 and 2 were converted to the estimated percentage of cotyledon surface area consumed and expressed as damaged and undamaged tissue proportions for analysis using generalized linear mixed‐effects models (GLMM) with a binomial error distribution. The proportion of damaged cotyledon tissue was modeled as a function of treatment (fixed effect = plant line), whereas seedling and seedling nested within arena were included as random effects. The final model was: glmer(cbind(damaged, undamaged) ∼ plant lines+(1∣arena/seedling), family = binomial). Models were fitted using the *glmer* function in the lme4 package.^53^ The significance of the fixed effects in the GLMM was determined by Wald ꭓ^2^ tests from the car package.^54^

Differences among seedling physiological traits (total glucosinolates, LEF, chlorophyll content and seedling biomass) were evaluated using one‐way analysis of variance (ANOVA) followed by Tukey's honestly significant difference (HSD) test for *post hoc* comparisons. Pearson's correlation coefficients were calculated to assess relationships between glucosinolate levels and other measured traits. To assess consistency of glucosinolate profiles across replicates, we conducted a PERMANOVA (adonis2) analysis in the vegan package.^55^ Bar graphs were generated using prism (v10; GraphPad Software LLC, Boston, MA, USA).

Non‐metric dimensional scaling (NMDS) was used to visualize the overall variation in glucosinolate profiles, feeding damage, seedling physiological traits (total glucosinolates, LEF, chlorophyll content and seedling biomass) across all six selected lines. Linear regression analyses were performed to evaluate the relationship between glucosinolates and *P. cruciferae* feeding damage. Additionally, the random forest analysis ranked gluconapin and total glucosinolates as the most important traits in predicting feeding damage of *P. cruciferae* among the variables examined. Chemical diversity indices were calculated using the chemodiv package.^56^ Diversity metrics were computed based on glucosinolate composition data for each replicate. Differences in chemical diversity among lines and its relationship with feeding damage were assessed using ANOVA and regression analyses. Comparison of flea beetle feeding damage between spring and summer experiments across 11 canola lines (Experiment 1) was done using Wilcoxon signed‐rank test.

## 3 RESULTS

### 3.1 Development of intergeneric introgression lines

The identified ILP markers (Table S1) were used to select *S. alba* genomic fragments in each generation while back‐crossing to N99‐508 to ensure that the *S. alba* insert was maintained. Markers with a presence/absence score between the *S. alba* and *B. napus* parents were utilized for screening. This resulted in introgression lines with six distinct *S. alba* introgressions (Table 1). To characterize the chromosomal inserts, all putative introgression lines were further tested with the KASP markers (Fig. S2). Specific attention was paid to alba chromosome regions known to be associated with both glucosinolate content and the fatty acid profile^46^ (as indicated by the blue and purple bars, respectively in Fig. S2). All lines appear to contain very large inserts and may indeed be whole chromosomes. Line VR19‐133 contains the *S. alba* chromosome SA01 (Fig. S2), lines VR19‐289 and VR21‐081 contain chromosome SA02, line VR19‐156 and BH14‐21772 contain chromosome SA03, line VR19‐176 contains chromosome SA05, whereas line VR19‐208 contains chromosome SA07. When screened with *S. alba* SA01 markers, only 3.5% of the lines within the DH population contained the introgression; however, when 24 seeds per DH line were selfed in these lines, the fragment was present in all progeny.

Interestingly chromosome SA02 has several known glucosinolate QTLs (Fig. S2) which would explain the increased glucosinolate content detected in VR19‐289. We had found previously that the *S. alba* introgression SA01 impacted the plant and seed phenotype of lines containing the introgression compared to those without it. For example, in preliminary field evaluations of the BH14‐21772 × YN01‐429 DH population over two seasons (2012 and 2013, 4 replicated plots per season), it was clear the presence of the SA01 introgression was reducing pod length (by 12 mm), lowered seed oil content (by 2%), had reduced emergence (by 6%) and altered the fatty acid profile (reducing oleic acid content by 3% while increasing linolenic by 3%) (unpublished data). The shift in fatty acid profiles was possibly caused by the presence of an *S. alba FAD2* allele present on SA01.^57^ Meanwhile, the SA01 introgression had no impact on seed protein and seed fiber content, seed color, seed glucosinolates, linolenic acid and erucic acid contents amongst the DH lines when assessed in the field (unpublished data).

### 3.2 Experiment 1 – feeding bioassay (11 lines)

Feeding damage by spring generation *P. cruciferae* varied significantly among Brassicaceae lines, indicating differences in host plant resistance [Fig. 1(A): GLMER: χ^2^(10) = 2300.1; *P* < 0.001]. Cotyledon feeding damage ranged from ≈12–55% across lines. Several introgressed lines, including VR19‐208, BH14‐21772, VR19‐289 and VR19‐156, sustained significantly lower feeding damage (<30%) compared to susceptible controls and the other introgressed lines (>38%). By contrast, the introgressed line VR19‐176 and *B. napus* parental line, N99‐508, exhibited the highest level of feeding damage (>50%).

Feeding damage caused by summer generation *P. cruciferae* also differed significantly among the lines [Fig. 1(B): GLMER: χ^2^(10) = 5528.3; *P* < 0.001], patterns of susceptibility were identical to those observed in the spring generation except VR19‐208 and Emergo. Unlike the spring generation, the summer generation did not cause elevated feeding damage on the *S. alba* parental line Emergo. However, VR19‐176 again exhibited consistently higher feeding damage (>60%) confirming its susceptibility across experiments. By contrast, introgressed lines BH14‐21772 and VR19‐289 maintained relatively low feeding damage in both the spring and summer bioassays (Fig. 1), suggesting stable resistance.

Comparison of feeding damage between spring and summer experiments using Wilcoxon signed‐rank tests showed that most lines did not differ significantly between experiments, suggesting generally consistent feeding responses across seasons. However, significant differences were observed for Emergo and VR19‐208 (*P* < 0.05), whereas the remaining lines showed no significant seasonal differences (Table S2).

### 3.3 Experiment 2 – feeding bioassays (six lines)

Feeding damage by spring generation *P. cruciferae* exhibited significant variation across the six selected Brassicaceae lines [Fig. 2(A): GLMER: χ^2^(5) = 1090.8; *P* < 0.001]. The parental line Emergo and introgressed lines BH14‐21772 and VR19‐289, consistently experienced the lowest percentage of damage, with Emergo demonstrating the minimal feeding damage overall. Similar results were observed in Experiment 1 bioassays on spring and summer generations. Intermediate levels of feeding were recorded for parental line N99‐508. Conversely, the susceptible introgressed lines, VR19‐208 and VR19‐176, incurred the highest feeding damage [Fig. 2(A)]. These results indicate that resistance responses were reproducible across experiments and not driven by trial‐specific variation.

**Figure 2:**
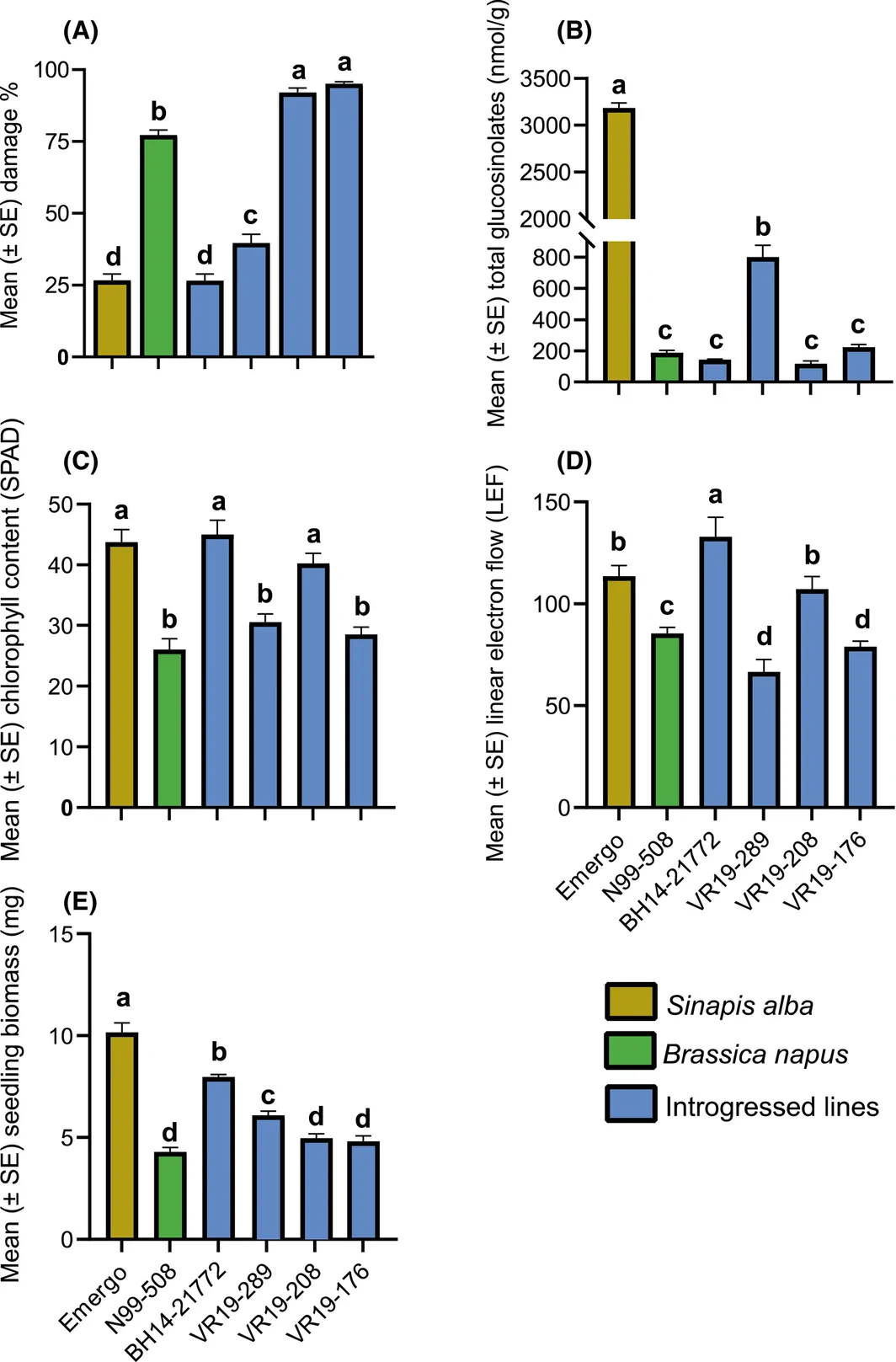
Comparison of six selected introgressed lines and controls (*B. napus* var. N99‐508, *S. alba* var. Emergo) for *P. cruciferae* (A) feeding damage (*n* = 10), (B) total glucosinolate concentrations (*n* = 5), (C) chlorophyll content (*n* = 10), (D) LEF (*n* = 10) and (E) seedling dry biomass (*n* = 10).

### 3.4 Glucosinolate content

Fifteen individual glucosinolates were detected, with substantial variation in both total concentration and composition across the introgressed lines (Table 2). Total glucosinolate concentrations differed significantly among lines [Fig. 2(B): *F*_(5,24)_ = 2012.10, *P* < 0.001], ranging from low levels in susceptible *B. napus* and introgressed lines to substantially higher levels in the *S. alba* parental line Emergo and select introgressed lines (Table 2). However, sinalbin was the dominant glucosinolate in the *S. alba* line Emergo and contributed substantially to its total glucosinolate content. Among individual compounds, gluconapin exhibited pronounced variation among lines and was present at elevated concentrations in Emergo andBH14‐21 772, and compared with the remaining lines. VR19‐289 contained approximately three‐fold higher concentrations of glucoalyssin, progoitrin, glucoraphanin and glucoerucin compared to the parental and other introgressed lines, and this line also exhibited significantly reduced flea beetle feeding damage (Table 2). By contrast, several glucosinolates were detected at low concentrations or were absent in some introgressed and parental lines. The PERMANOVA results showed that glucosinolates composition differed significantly among lines (*P* < 0.001), whereas replicate had no significant effect (*P* = 0.355), indicating that glucosinolate profiles were consistent across experimental replicates.

**Table 2:** Glucosinolates concentration (nmol g^–1^ DW, mean ± SE) in different Brassicaceae lines.

Glucosinolates	*S. alba*	*B. napus*	Introgressed lines (*S. alba* × *B. napus*)			
	Emergo	N99‐508	BH14‐21772	VR19‐289	VR19‐208	VR19‐176
Glucohirsutin	7.09 ± 0.10	3.26 ± 0.19	1.42 ± 0.02	10.54 ± 0.77	2.95 ± 0.14	5.70 ± 0.57
4‐Hydroxyglucobrassicin	0.71 ± 0.03	1.96 ± 0.04	1.57 ± 0.09	4.18 ± 0.39	1.08 ± 0.09	1.98 ± 0.20
Glucocapparin	291.5 ± 7.33	104.23 ± 2.07	88.64 ± 1.42	150.8 ± 3.24	91.9 ± 5.95	29.15 ± 4.14
Glucoarabin	0.07 ± 0.00	0.06 ± 0.00	0.04 ± 0.00	0.14 ± 0.01	0.05 ± 0.00	0.08 ± 0.01
Glucoraphenin	0.04 ± 0.01	0.22 ± 0.02	0.17 ± 0.01	0.38 ± 0.01	0.21 ± 0.02	0.21 ± 0.03
Glucoalyssin	0.91 ± 0.03	22.16 ± 1.67	18.72 ± 0.78	330.89 ± 10.55	10.18 ± 0.72	95.06 ± 3.27
Epiprogoitrin	8.13 ± 0.32	1.31 ± 0.06	0.47 ± 0.03	2.47 ± 0.19	0.14 ± 0.01	1.052 ± 0.10
Glucoerucin	0.96 ± 0.03	0.14 ± 0.01	0.27 ± 0.00	5.86 ± 0.20	0.21 ± 0.02	1.23 ± 0.14
Glucoiberin	1.78 ± 0.11	2.21 ± 0.01	1.51 ± 0.10	3.38 ± 0.18	2.44 ± 0.44	2.29 ± 0.11
Progoitrin	4.69 ± 0.33	44.83 ± 2.70	20.52 ± 0.35	189.63 ± 14.19	6.59 ± 0.43	56.64 ± 4.09
Sinalbin	2860.62 ± 48	0.48 ± 0.01	0.25 ± 0.04	0.52 ± 0.04	0.31 ± 0.03	3.45 ± 0.49
Sinigrin	0.14 ± 0.01	0.03 ± 0.01	0.02 ± 0.00	0.04 ± 0.00	0.03 ± 0.00	0.03 ± 0.00
Glucoraphanin	1.27 ± 0.06	0.82 ± 0.11	0.71 ± 0.10	100.66 ± 7.51	0.69 ± 0.05	24.41 ± 1.33
Glucotropaeolin	4.65 ± 0.15	0.43 ± 0.02	0.28 ± 0.01	1.24 ± 0.07	0.17 ± 0.01	0.68 ± 0.64
Gluconapin	2.53 ± 0.19	6.81 ± 0.35	9.62 ± 0.21	0.58 ± 0.03	0.31 ± 0.02	0.41 ± 0.05
Total glucosinolates	3185.0 ± 55.1	188.9 ± 6.2	144.2 ± 6.2	801.3 ± 33.7	117.2 ± 2.7	222.3 ± 7.9

### 3.5 Chlorophyll content and linear electron flow

Chlorophyll content in cotyledons varied significantly among the Brassicaceae lines examined [Fig. 2(C): *F*_(5,54)_ = 21.4, *P* < 0.001]. The introgressed lines, BH14‐21772 and VR19‐289, along with the *S. alba* parental line Emergo, exhibited substantially higher chlorophyll levels (≈40–45 SPAD) compared to the remaining lines (25–30 SPAD).

The LEF showed a similar pattern of significant variation among the lines [Fig. 2(D): *F*_(5,54)_ = 17.23, *P* < 0.001]. BH14‐21772 and VR19‐289, and Emergo exhibited substantially higher LEF values (110–130 μmol electrons m^−2^ s^−1^), whereas all other lines recorded lower LEF values (60–80 μmol electrons m^−2^ s^−1^).

### 3.6 Seedling biomass

Above‐ground dry biomass differed significantly among all lines tested [Fig. 2(E): *F*_(5,54)_ = 69.59, *P* < 0.001]. The *S. alba* parental line Emergo had the highest dry biomass (~10 mg), followed by BH14‐21772 (~8 mg) and VR19‐289 (~6 mg); these three lines were significantly different from each other. However, the three remaining lines had significantly lower dry biomass (~ 5 mg) and were not significantly different from one another [Fig. 2(E)].

### 3.7 Trichrome density and length

Among the six Brassicaceae lines evaluated, trichomes were only observed on the *S. alba* Emergo parental line (Fig. S3) and were absent in all *B. napus* and introgressed lines. Trichome measurements are presented here as a descriptive trait of the parental line, as no direct relationship between trichome characteristics and feeding damage was assessed in this study. The trichome length on the abaxial cotyledon surface was 60 ± 3 μm [Fig. S2(A)], 45 ± 2 μm on the adaxial surface and on hypocotyls was 92 ± 7 μm. The trichome density was 340 ± 9 mm^–2^ on the abaxial cotyledon surface [Fig. S3(A),(D)],15 ± 0.8 mm^–2^ on the adaxial surface [Fig. S3(B)] and 301 ± 11 mm^–2^ on hypocotyls [Fig. S3(E)].

### 3.8 Relationship between glucosinolates and P. Cruciferae feeding damage

Pearson correlation analysis revealed total glucosinolate concentration was significantly correlated with only five glucosinolates of the 15 glucosinolates examined [Fig. 3(A); Table S3]. Gluconapin was not positively correlated with any other glucosinolates quantified [Fig. 3(A); Table S3], indicating partial independence among glucosinolates. Based on these results, gluconapin and total glucosinolates were selected for further regression analysis.

**Figure 3:**
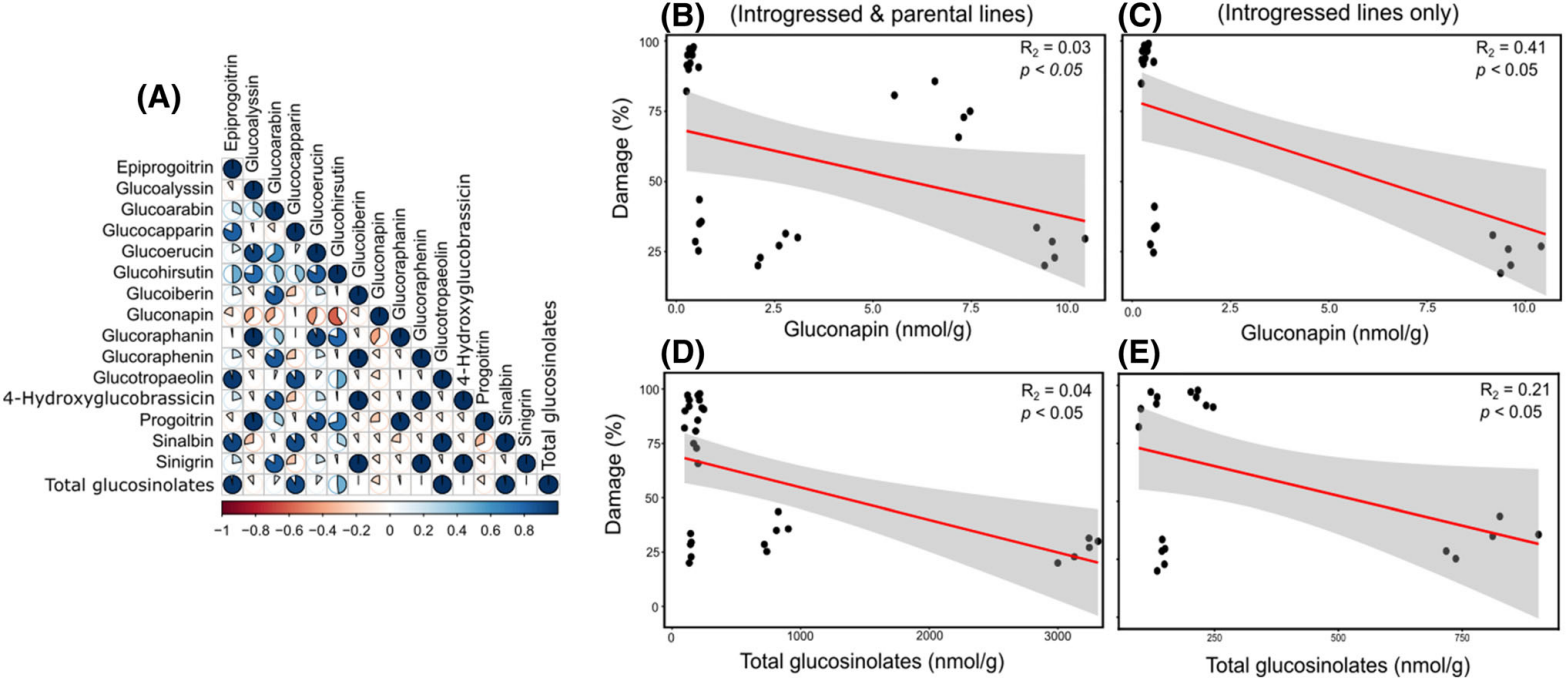
Correlation matrix of glucosinolates concentrations (nmol g^–1^ DW) of Brassicaceae lines (A), relationship between gluconapin concentration and *P. cruciferae* feeding damage for introgressed and parental lines (B) and introgressed lines only (C), and relationship between total glucosinolate concentration and *P. cruciferae* feeding damage for introgressed and parental lines (D) and introgressed lines only (E). In (A), darker blue and red pie charts indicate stronger positive and negative correlation coefficients (*r*), respectively. Solid red lines represent linear regression lines, and gray shading indicates the 95% CI (B–E).

Linear regression analyses demonstrated that increasing concentrations of both gluconapin and total glucosinolates were correlated with reduced *P. cruciferae* feeding damage among the introgressed and parental lines [Fig. 3(B),(D)]. This association remained consistent when the parental lines Emergo and N99‐508 were excluded from the analysis [Fig. 3(C),(E)]. Chemical diversity indices based on glucosinolates differed among lines [*P* < 0.05: Fig. S4(A)]; however, they were not significantly correlated with flea beetle feeding damage [*P* > 0.05: Fig. S4(B)]. Additionally, the random forest analysis ranked gluconapin and total glucosinolates as the most important traits in predicting feeding damage of *P. cruciferae* among the variables examined (Fig. S5). The random forest model was constructed using 500 trees with five variables randomly selected at each split. The model produced an out‐of‐bag (OOB) mean squared error of 1.6 and explained 82.7% of the variance in *P. cruciferae* feeding damage, indicating strong predictive ability of the selected variables across the tested lines.

### 3.9 Multivariate relationships among plant traits and feeding damage

The NMDS ordination revealed clear separation among Brassicaceae lines based on glucosinolate profiles, physiological traits and feeding damage (Fig. 4). All lines were separated in the ordination space, with VR19‐289 and Emergo positioned closer to vectors representing chlorophyll content, LEF and seedling biomass, whereas BH14‐21772 aligned with the gluconapin vector. By contrast, VR19‐208 and VR19‐176 were located in the direction of higher *P. cruciferae* feeding damage. Vectors representing total glucosinolate content were oriented away from feeding damage and toward lines exhibiting reduced damage. The glucosinolate composition of Emergo was distinct from other genotypes because it clustered with glucoraphanin, gluconasturtiin and glucoraphenin. The ordination results demonstrate that each genotype has unique chemical and physiological characteristics which may explain its resistance patterns.

**Figure 4:**
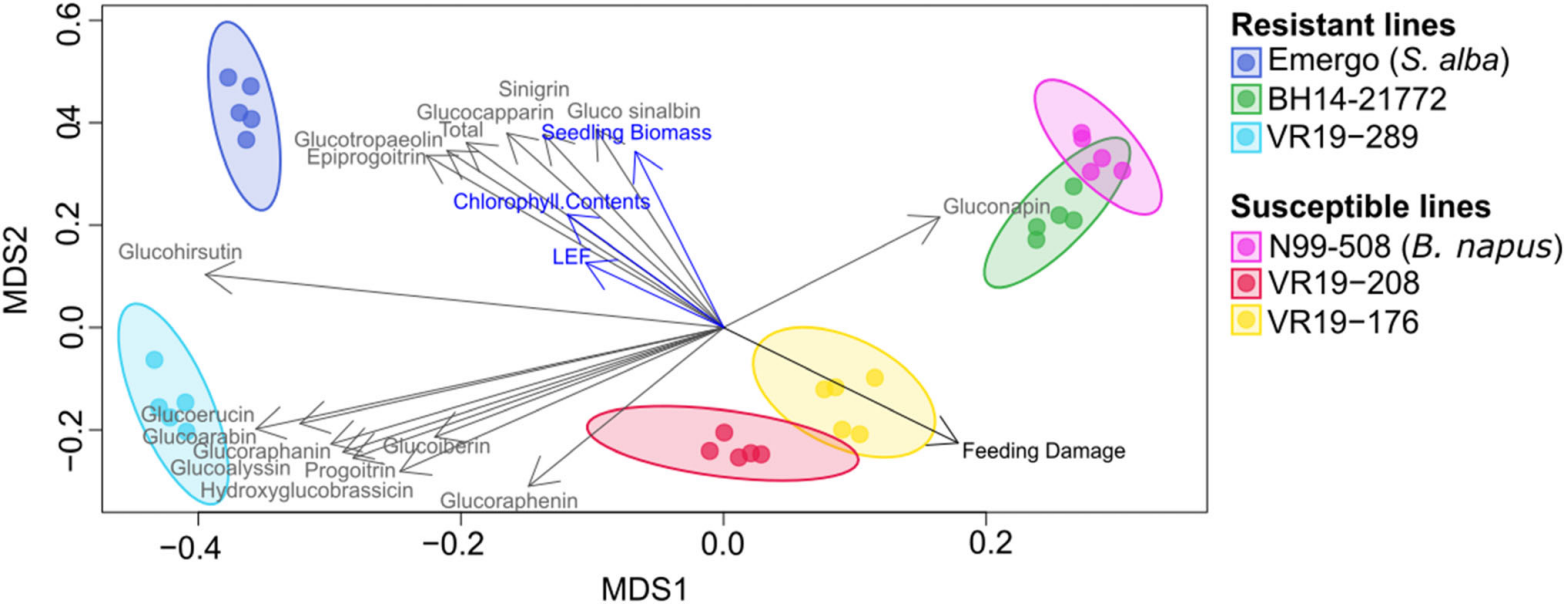
Effects of Brassicaceae lines with different glucosinolate profiles on *P. cruciferae* damage. Concentrations of individual glucosinolates (nmol g^–1^ DW) were used in the analysis. Data were analyzed by gradient analysis using NMDS. Different colored ellipses represent the six Brassicaceae lines tested in the bioassay. Gray vectors represent different glucosinolates, blue vectors represent seedling physiological parameters and black vectors represnt *P. cruciferae* damage.

## 4 DISCUSSION

This study demonstrates variation in susceptibility to *P. cruciferae* among *B. napus* germplasm with *S. alba* introgressions, with resistance associated with variation in glucosinolate profiles and seedling physiological traits. Across multiple feeding bioassays and flea beetle generations, introgressed lines generally exhibited reproducible differences in susceptibility; however, some lines (e.g. VR19‐208) showed variability between experiments, indicating that resistance expression may be influenced by different beetle generations. Lines with elevated total glucosinolate concentrations, particularly gluconapin, generally sustained lower feeding damage, suggesting that chemical defenses may contribute to flea beetle–host interactions.

Glucosinolates are well‐established mediators of plant defense in the Brassicaceae, influencing host selection and feeding behavior of specialists insect herbivores including *Phyllotreta* spp.^24^, ^58^, ^59^, ^60^ Previous work has shown that *S. alba* exhibits reduced flea beetle feeding relative to *B. napus*, often attributed to glucosinolate profiles and rapid early growth.^28^, ^29^ Our findings are consistent with these results as the *S. alba* parental line Emergo sustained minimal feeding damage (in two of three experiments) and exhibited the highest total glucosinolate concentrations. Importantly, introgressed lines BH14‐21772 and VR19‐289, which exhibited either elevated total glucosinolate levels or distinct glucosinolate profiles, also showed reduced feeding damage. These results suggest that resistance‐associated traits derived from *S. alba* can be expressed within the *B. napus* background, although the specific compounds involved may vary among lines. However, the relationship between glucosinolates and feeding is complex, as specialist herbivores such as *P. cruciferae* may respond differently to specific glucosinolate compounds or glucosinolate profiles. Likewise, Rüde *et al*.^61^ reported that cotyledon‐stage resistance in *S. alba* against *Psylliodes chrysocephala* L. (Coleoptera: Chrysomelidae) was associated with distinct glucosinolate and metabolite profiles, including elevated sinalbin concentrations and deterrent effects of sinapinic acid. Their findings further support the importance of seedling‐stage chemical defenses in shaping flea beetle feeding behavior and highlight that resistance may involve multiple interacting metabolites rather than total glucosinolate concentration alone.

These results collectively offer support to the hypothesis that the introgressed lines acquired resistance to *P. cruciferae* via *S. alba* genomic introgressions and supports value in this approach for breeding canola for flea beetle resistance. However, the commercial potential of these introgressed lines remains to be determined; *S. alba* is historically characterized by significantly lower oil content and inferior seed meal quality compared to *B. napus*.^62^ Glucosinolate content in seeds is a major determinant of canola oil and meal quality, and elevated concentrations are undesirable for human and animal consumption. In the present study, glucosinolates were quantified only in cotyledon tissues, and their relationship with seed glucosinolate content was not assessed; therefore, caution is required when extrapolating these findings to seed quality traits. Although glucosinolate concentrations observed in cotyledons were relatively low, further evaluation is required to determine whether resistance‐associated traits can be incorporated without introducing linkage drag or compromising the low seed glucosinolate thresholds required for modern canola quality standards.^63^

Among individual compounds, gluconapin emerged as a strong predictor of reduced feeding damage. Regression and random forest analysis confirmed that gluconapin and total glucosinolate concentration as the most influential biochemical traits measured associated with resistance and may support their use as biochemical markers in resistance screening. Prior studies have emphasized sinalbin as a key deterrent in *S. alba*,^24^, ^27^ contributing substantially to total glucosinolate levels in Emergo. This has been recently supported by Giguère *et al*.,^45^ who demonstrated that sinalbin acts as a feeding deterrent against *P. chrysocephala* in *S. alba* using bioassay‐guided fractionation. However, the introgressed lines evaluated in this study did not exhibit elevated sinalbin levels, suggesting that resistance‐associated variation may instead be mediated by differences in other glucosinolates or overall chemical composition. These results underscore the importance of evaluating both total glucosinolate content and individual compounds when screening for resistance, rather than focusing on single metabolites alone.

In addition to chemical traits, lines with lower feeding damage (Emergo, BH14‐21772 and VR19‐289) exhibited enhanced physiological performance, including higher chlorophyll content, higher LEF and increased biomass compared to more susceptible lines. LEF is a commonly used photosynthetic parameter that reflects the rate of electron transport during photosynthesis.^64^ These physiological traits were associated with reduced feeding damage and elevated glucosinolate concentrations, suggesting that some resistant lines were able to maintain chemical defenses without obvious reductions in early seedling growth. This contrasts with findings in the model Brassicaceae *Arabidopsis thaliana* L. (Heynh.), where glucosinolate production may increase photosynthetic demands and reduce biomass accumulation.^65^ However, the relationship between photosynthetic performance, growth and glucosinolate production remains complex, and no causal relationship can be inferred from the present study.

Trichomes were observed only in the *S. alba* line Emergo, and therefore their independent role in resistance could not be directly assessed. However, the introgressed line BH14‐21772, which lacked trichomes and had lower total glucosinolate levels compared to Emergo, exhibited similar feeding damage, suggesting that trichomes are unlikely to be the primary factor influencing resistance among the lines tested. Previous work, using transgenic *B. napus* overexpressing trichomes found reduced flea beetle feeding, but cotyledons were still glabrous (no trichomes), and reduced feeding was found mainly on the stems, petioles, and first true leaves.^49^ In the future, development of *B. napus* with increased trichomes and chemical defenses would be beneficial to explore and capitalize on.

Spring and summer generations of *P. cruciferae* differ in their feeding damage on canola.^17^ Here, resistance patterns were generally consistent across the two generations, and particularly promising are the introgressed lines BH14‐21772 and VR19‐289 which consistently showed reduced feeding damage across all generations and experiments. This stability across generations offers empirical evidence that resistance traits in introgressed lines may exhibit long‐term durability, a conclusion not thoroughly established in prior research.^17^, ^20^, ^66^

Multivariate ordination further supported a multitrait basis for resistance, with lines clustering according to combined chemical and physiological characteristics rather than single traits alone. This patterns supports the concept of ‘resistance syndromes,’ where synergistic combinations of traits enhance plant defense effectiveness.^67^, ^68^ For canola breeding, this emphasizes the value of selecting complementary trait combinations (e.g. glucosinolate levels plus physiological vigor) rather than focusing on single defense traits.

Several limitations of this study should be acknowledged. First, we quantified constitutive glucosinolate levels but did not assess induced responses triggered by flea beetle feeding. Induced glucosinolates production has been reported in Brassicaceae, although its role in *P. cruciferae* resistance remains ambiguous.^28^, ^29^ Second, the nutritional and biochemical quality of Brassicaceae seedlings extends beyond glucosinolates alone. Primary metabolites such as nitrogen and sugars, as well as physical traits like leaf toughness, may also influence feeding preferences.^69^ Third, genotype × environment interactions should be considered. Resistance observed under controlled conditions may vary under drought, nutrient stress, or different soil types, which could alter glucosinolate expression and *P. cruciferae* feeding behavior.^70^, ^71^ Finally, interactions with natural enemies^72^, ^73^ and landscape‐level factors,^74^, ^75^ which can substantially affect flea beetle populations, were not addressed and warrant further investigation.

From an applied perspective, our findings suggest that resistant introgressed lines such as BH14‐21772 and VR19‐289 provide valuable material to identify resistance mechanisms, and with further breeding efforts, could be incorporated into elite canola varieties to reduce early‐season feeding pressure and enhance the consistency of control when used alongside existing cultural and chemical tactics. By lowering the intensity of flea beetle injury during the vulnerable seedling stage, such lines may reduce the need for reactive foliar insecticide applications and contribute to more resilient, sustainable canola production systems.

From a genetic perspective, variation in resistance among introgressed lines also highlights the importance of genomic background and introgression structure. For example, BH14‐21772 and VR19‐156 both carry introgressions derived from the same *S. alba* chromosome (SA03) and originated from a common BC_2_ line, but were developed using different backcross parents. This suggests that background genetics, likely in combination with the introgressed genomic content, may influence resistance expression.^76^ Additionally, independent recombination events may have altered the size, composition, or chromosomal positioning of the SA03‐derived fragment within the *B. napus* genome, potentially contributing to phenotypic differences.^77^

Likewise, VR19‐289 (resistant) and VR21‐081 (susceptible) both contain introgressions from *S. alba*, but from different donor sources. Notably, Emergo—the *S. alba* source for VR21‐081—is itself highly resistant, yet this resistance does not appear to be conferred by genes within the introgressed segment in that line. Together, these contrasts indicate that resistance is not determined solely by the presence of an introgression, but may depend on the specific genomic context, recombination history, and interactions between introgressed and recipient genome regions.^78^, ^79^ The outcrossing‐nature of *S. alba* that leads to heterogeneity within cultivars of this species should also be considered in the development of the introgression lines. Because of this, the genetic content introgressed into *B. napus* may not be identical to original *S. alba* parents assessed in this study, or valuable alleles may have not been fixed in the introgression lines.

## 5 CONCLUSIONS

In conclusion, this study provides evidence that resistance to *P. cruciferae* in introgressed *B. napus* × *S. alba* germplasm is mediated primarily by glucosinolate‐associated chemical defenses, supported by strong seedling physiological performance. The identification of total glucosinolates and gluconapin as key resistance‐associated traits offers practical biomarkers for resistance screening and breeding. Together, these findings advance understanding of Brassicaceae–flea beetle interactions and provide a foundation for integrating host plant resistance into next‐generation IPM strategies for canola.

## CONFLICT OF INTEREST

The authors have no conflicts of interest to disclose.

## Supporting information


**Figure S1.** Crossing schemes for the two intergeneric pedigrees. Lines highlighted in green are *B. napus*, in purple are *S. alba* and in yellow are the selected *B. napus* lines with stable *S. alba* inserts. Generations highlighted in orange represent the stages that were screened to isolate lines with detected inserts. The red box displays the portion of the pedigree required to stabilize the genome and to consistently maintain the *S. alba* insert.
**Figure S3.** *Sinapis alba* line Emergo showing trichome density on (A) abaxial and (B) adaxial surfaces. The small red arrow in (A) and (B) indicates the trichome. Color images showing trichome development on the (C) seedling, (D) abaxial cotyledon and (E) hypocotyls.
**Figure S4.** Variation in glucosinolate chemical diversity (Shannon index) among Brassicaceae lines (A) and its association with *P. cruciferae* feeding damage (B).
**Figure S5.** Variable importance from random forest analysis of glucosinolate profiles from six selected Brassicaceae lines using *P. cruciferae* feeding damage ratings.
**Table S1.** ILP primers utilized for selection during back cross generations
**Table S2.** Comparison of flea beetle feeding damage between spring and summer experiments across 11 canola lines using Wilcoxon signed‐rank tests. ns, not significant (*P* > 0.05)
**Table S3.** Pearson correlation degree of significance (*P*‐value) for individual glucosinolates concentration (nmol g^–1^ DW) in different Brassicaceae lines used in the Experiment 2. Bolded *P*‐values are significant at α = 0.05
**Table S4.** Classification and side‐chain profiles of selected glucosinolates in different Brassicaceae lines.

**Figure S2:** Sinapisalba genetic map and QTLs associated with fatty acid and glucosinolatecontent as per Javidfar and Cheng (2013) and Zeng *et al*. (2017). Purple (fatty acid) and blue bars (glucosinolate: Table S4) represent the location of QTLs associated with the listed traits. Markers in blue represent additional KASP markers added via a GBS analysis. Markers in bold green represent the markers present in the specific introgression line listed above the chromosome. Markers in orange amplify in *B. napus* and thus cannot be used to confirm the presence of the *S. alba* insert.

## Data Availability

The data that support the findings of this study are available on request from the corresponding author. The data are not publicly available due to privacy or ethical restrictions.
