# Supplementary material for: Introgressed Brassica napus × Sinapis alba enhance resistance to the crucifer flea beetle (Phyllotreta cruciferae)
Source: Pest Manag Sci. 2026 Jul 14;82(11):10752–64. doi: 10.1002/ps.71097 (PMC13665644; doi:10.1002/ps.71097)
Supplement: Supplementary file 1 [file PS-82-10752-s001.pdf]

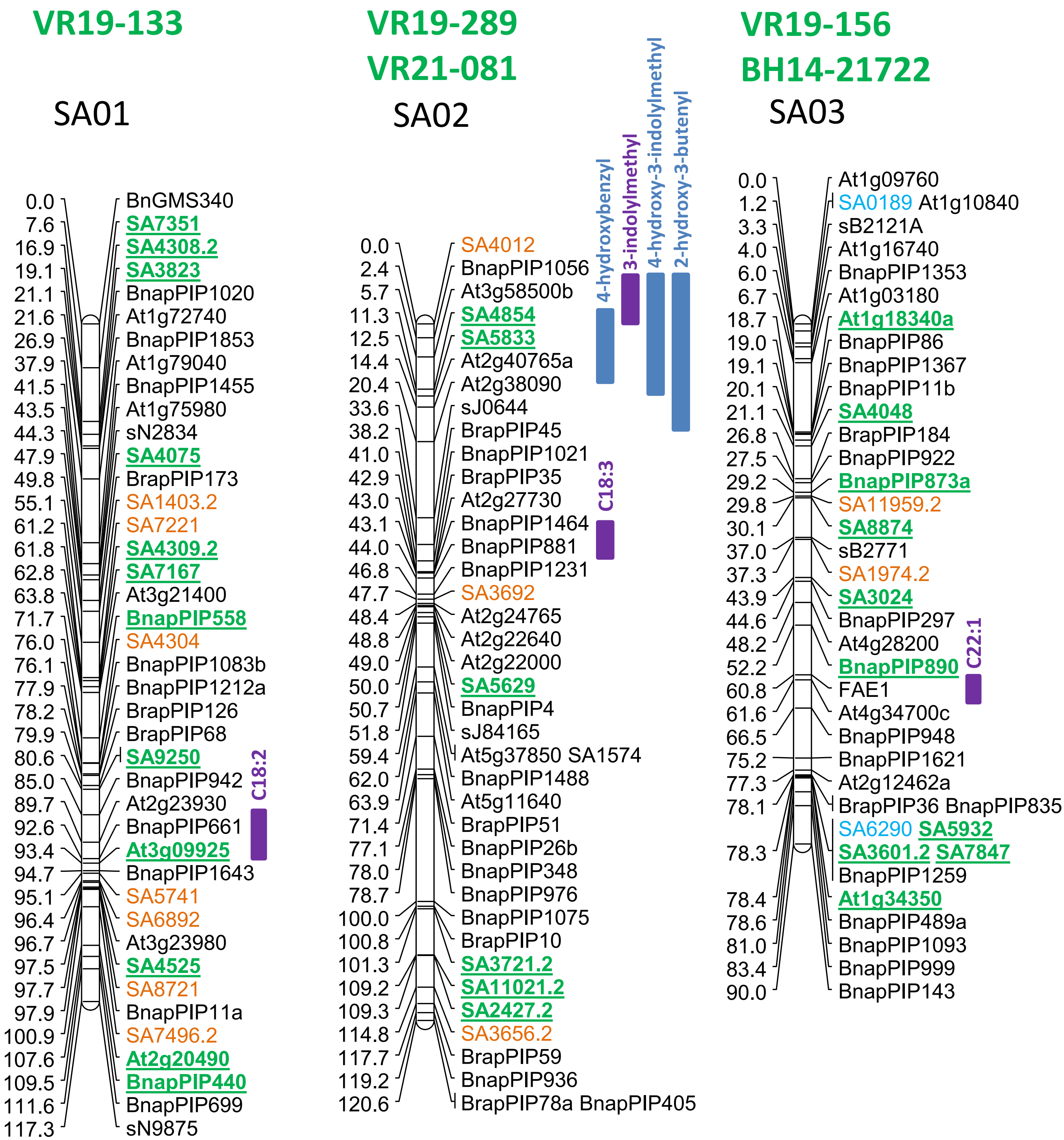
VR19-133
SA01
0.0 BnGMS340
7.6 SA7351
16.9 SA4308.2
19.1 SA3823
21.1 BnapPIP1020
21.6 At1g72740
26.9 BnapPIP1853
37.9 At1g79040
41.5 BnapPIP1455
43.5 At1g75980
44.3 sN2834
47.9 SA4075
49.8 BrapPIP173
55.1 SA1403.2
61.2 SA7221
61.8 SA4309.2
62.8 SA7167
63.8 At3g21400
71.7 BnapPIP558
76.0 SA4304
76.1 BnapPIP1083b
77.9 BnapPIP1212a
78.2 BrapPIP126
79.9 BrapPIP68
80.6 SA9250
85.0 BnapPIP942
89.7 At2g23930
92.6 BnapPIP661
93.4 At3g09925
94.7 BnapPIP1643
95.1 SA5741
96.4 SA6892
96.7 At3g23980
97.5 SA4525
97.7 SA8721
97.9 BnapPIP11a
100.9 SA7496.2
107.6 At2g20490
109.5 BnapPIP440
111.6 BnapPIP699
117.3 sN9875
C18:2
VR19-289
VR21-081
SA02
0.0 SA4012
2.4 BnapPIP1056
5.7 At3g58500b
11.3 SA4854
12.5 SA5833
14.4 At2g40765a
20.4 At2g38090
33.6 sJ0644
38.2 BrapPIP45
41.0 BnapPIP1021
42.9 BrapPIP35
43.0 At2g27730
43.1 BnapPIP1464
44.0 BnapPIP881
46.8 BnapPIP1231
47.7 SA3692
48.4 At2g24765
48.8 At2g22640
49.0 At2g22000
50.0 SA5629
50.7 BnapPIP4
51.8 sJ84165
59.4 At5g37850 SA1574
62.0 BnapPIP1488
63.9 At5g11640
71.4 BrapPIP51
77.1 BnapPIP26b
78.0 BnapPIP348
78.7 BnapPIP976
100.0 BnapPIP1075
100.8 BrapPIP10
101.3 SA3721.2
109.2 SA11021.2
109.3 SA2427.2
114.8 SA3656.2
117.7 BrapPIP59
119.2 BnapPIP936
120.6 BrapPIP78a BnapPIP405
4-hydroxybenzyl
3-indolylmethyl
4-hydroxy-3-indolylmethyl
2-hydroxy-3-butenyl
C18:3
VR19-156
BH14-21722
SA03
0.0 At1g09760
1.2 SA0189 At1g10840
3.3 sB2121A
4.0 At1g16740
6.0 BnapPIP1353
6.7 At1g03180
18.7 At1g18340a
19.0 BnapPIP86
19.1 BnapPIP1367
20.1 BnapPIP11b
21.1 SA4048
26.8 BrapPIP184
27.5 BnapPIP922
29.2 BnapPIP873a
29.8 SA11959.2
30.1 SA8874
37.0 sB2771
37.3 SA1974.2
43.9 SA3024
44.6 BnapPIP297
48.2 At4g28200
52.2 BnapPIP890
60.8 FAE1
61.6 At4g34700c
66.5 BnapPIP948
75.2 BnapPIP1621
77.3 At2g12462a
78.1 BrapPIP36 BnapPIP835
78.3 SA6290 SA5932
SA3601.2 SA7847
BnapPIP1259
78.4 At1g34350
78.6 BnapPIP489a
81.0 BnapPIP1093
83.4 BnapPIP999
90.0 BnapPIP143
C22:1

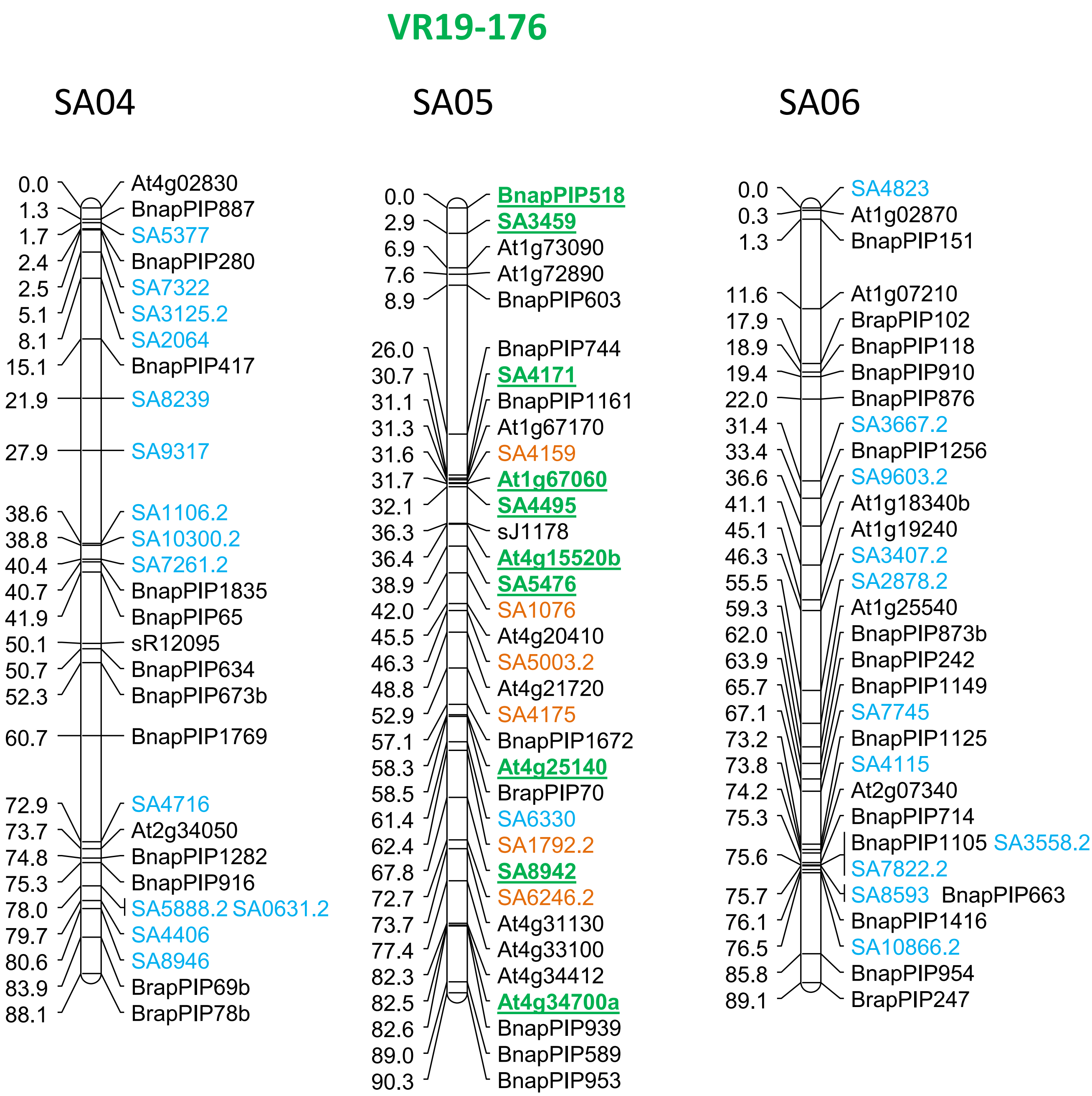
VR19-176
SA04
0.0 At4g02830
1.3 BnapPIP887
1.7 SA5377
2.4 BnapPIP280
2.5 SA7322
5.1 SA3125.2
8.1 SA2064
15.1 BnapPIP417
21.9 SA8239
27.9 SA9317
38.6 SA1106.2
38.8 SA10300.2
40.4 SA7261.2
40.7 BnapPIP1835
41.9 BnapPIP65
50.1 sR12095
50.7 BnapPIP634
52.3 BnapPIP673b
60.7 BnapPIP1769
72.9 SA4716
73.7 At2g34050
74.8 BnapPIP1282
75.3 BnapPIP916
78.0 SA5888.2 SA0631.2
79.7 SA4406
80.6 SA8946
83.9 BrapPIP69b
88.1 BrapPIP78b
SA05
0.0 BnapPIP518
2.9 SA3459
6.9 At1g73090
7.6 At1g72890
8.9 BnapPIP603
26.0 BnapPIP744
30.7 SA4171
31.1 BnapPIP1161
31.3 At1g67170
31.6 SA4159
31.7 At1g67060
32.1 SA4495
36.3 sJ1178
36.4 At4g15520b
38.9 SA5476
42.0 SA1076
45.5 At4g20410
46.3 SA5003.2
48.8 At4g21720
52.9 SA4175
57.1 BnapPIP1672
58.3 At4g25140
58.5 BrapPIP70
61.4 SA6330
62.4 SA1792.2
67.8 SA8942
72.7 SA6246.2
73.7 At4g31130
77.4 At4g33100
82.3 At4g34412
82.5 At4g34700a
82.6 BnapPIP939
89.0 BnapPIP589
90.3 BnapPIP953
SA06
0.0 SA4823
0.3 At1g02870
1.3 BnapPIP151
11.6 At1g07210
17.9 BrapPIP102
18.9 BnapPIP118
19.4 BnapPIP910
22.0 BnapPIP876
31.4 SA3667.2
33.4 BnapPIP1256
36.6 SA9603.2
41.1 At1g18340b
45.1 At1g19240
46.3 SA3407.2
55.5 SA2878.2
59.3 At1g25540
62.0 BnapPIP873b
63.9 BnapPIP242
65.7 BnapPIP1149
67.1 SA7745
73.2 BnapPIP1125
73.8 SA4115
74.2 At2g07340
75.3 BnapPIP714
75.6 BnapPIP1105 SA3558.2 SA7822.2 SA8593 BnapPIP663
75.7 BnapPIP1416
76.1 SA10866.2
76.5 BnapPIP954
85.8 BrapPIP247
89.1

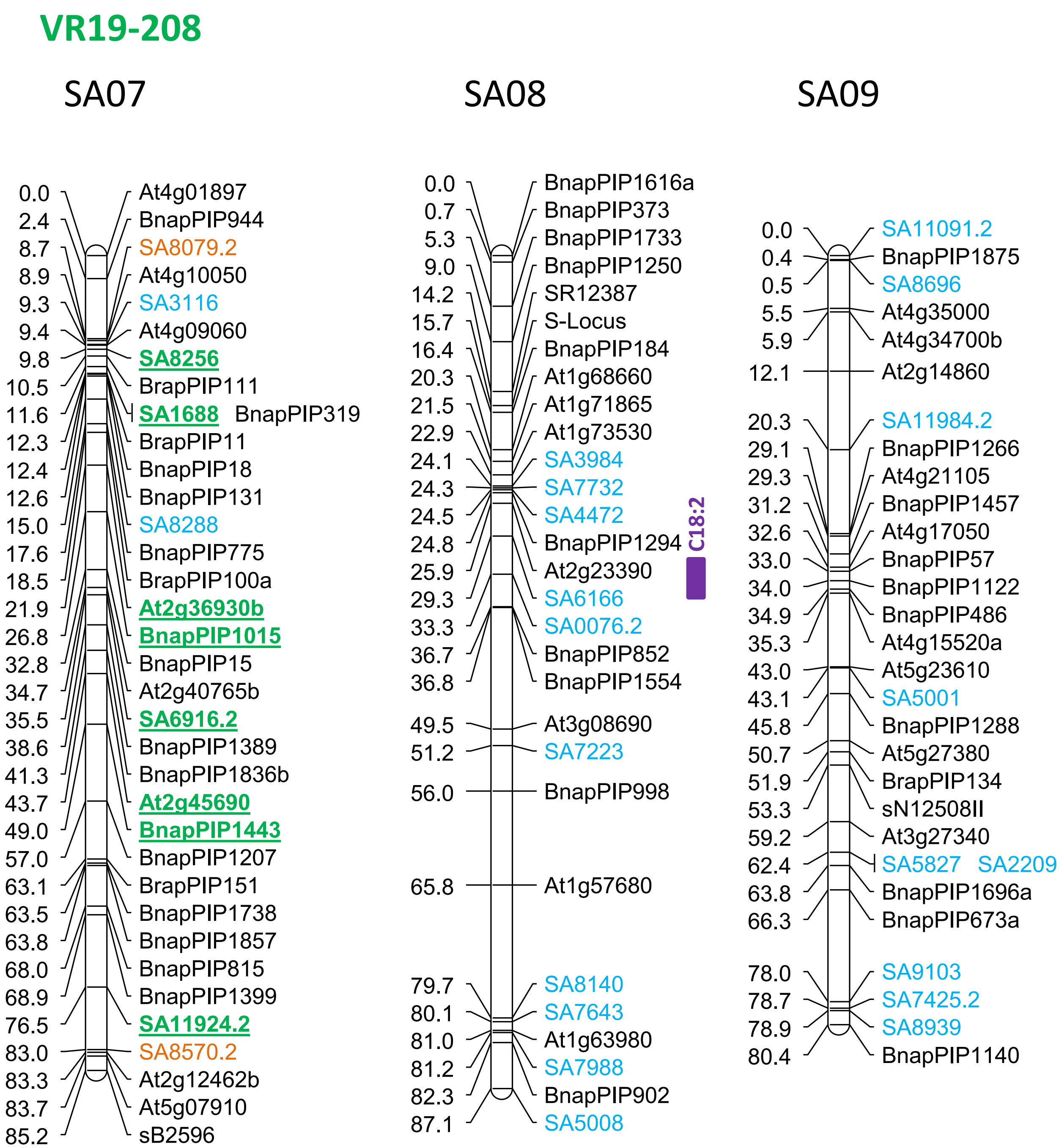
VR19-208
SA07
0.0 At4g01897
2.4 BnapPIP944
8.7 SA8079.2
8.9 At4g10050
9.3 SA3116
9.4 At4g09060
9.8 SA8256
10.5 BrapPIP111
11.6 SA1688 BnapPIP319
12.3 BrapPIP11
12.4 BnapPIP18
12.6 BnapPIP131
15.0 SA8288
17.6 BnapPIP775
18.5 BrapPIP100a
21.9 At2g36930b
26.8 BnapPIP1015
32.8 BnapPIP15
34.7 At2g40765b
35.5 SA6916.2
38.6 BnapPIP1389
41.3 BnapPIP1836b
43.7 At2g45690
49.0 BnapPIP1443
57.0 BnapPIP1207
63.1 BrapPIP151
63.5 BnapPIP1738
63.8 BnapPIP1857
68.0 BnapPIP815
68.9 BnapPIP1399
76.5 SA11924.2
83.0 SA8570.2
83.3 At2g12462b
83.7 At5g07910
85.2 sB2596
SA08
0.0 BnapPIP1616a
0.7 BnapPIP373
5.3 BnapPIP1733
9.0 BnapPIP1250
14.2 SR12387
15.7 S-Locus
16.4 BnapPIP184
20.3 At1g68660
21.5 At1g71865
22.9 At1g73530
24.1 SA3984
24.3 SA7732
24.5 SA4472
24.8 BnapPIP1294
25.9 At2g23390
29.3 SA6166
33.3 SA0076.2
36.7 BnapPIP852
36.8 BnapPIP1554
49.5 At3g08690
51.2 SA7223
56.0 BnapPIP998
65.8 At1g57680
79.7 SA8140
80.1 SA7643
81.0 At1g63980
81.2 SA7988
82.3 BnapPIP902
87.1 SA5008
C18:2
SA09
0.0 SA11091.2
0.4 BnapPIP1875
0.5 SA8696
5.5 At4g35000
5.9 At4g34700b
12.1 At2g14860
20.3 SA11984.2
29.1 BnapPIP1266
29.3 At4g21105
31.2 BnapPIP1457
32.6 At4g17050
33.0 BnapPIP57
34.0 BnapPIP1122
34.9 BnapPIP486
35.3 At4g15520a
43.0 At5g23610
43.1 SA5001
45.8 BnapPIP1288
50.7 At5g27380
51.9 BrapPIP134
53.3 sN12508II
59.2 At3g27340
62.4 SA5827 SA2209
63.8 BnapPIP1696a
66.3 BnapPIP673a
78.0 SA9103
78.7 SA7425.2
78.9 SA8939
80.4 BnapPIP1140

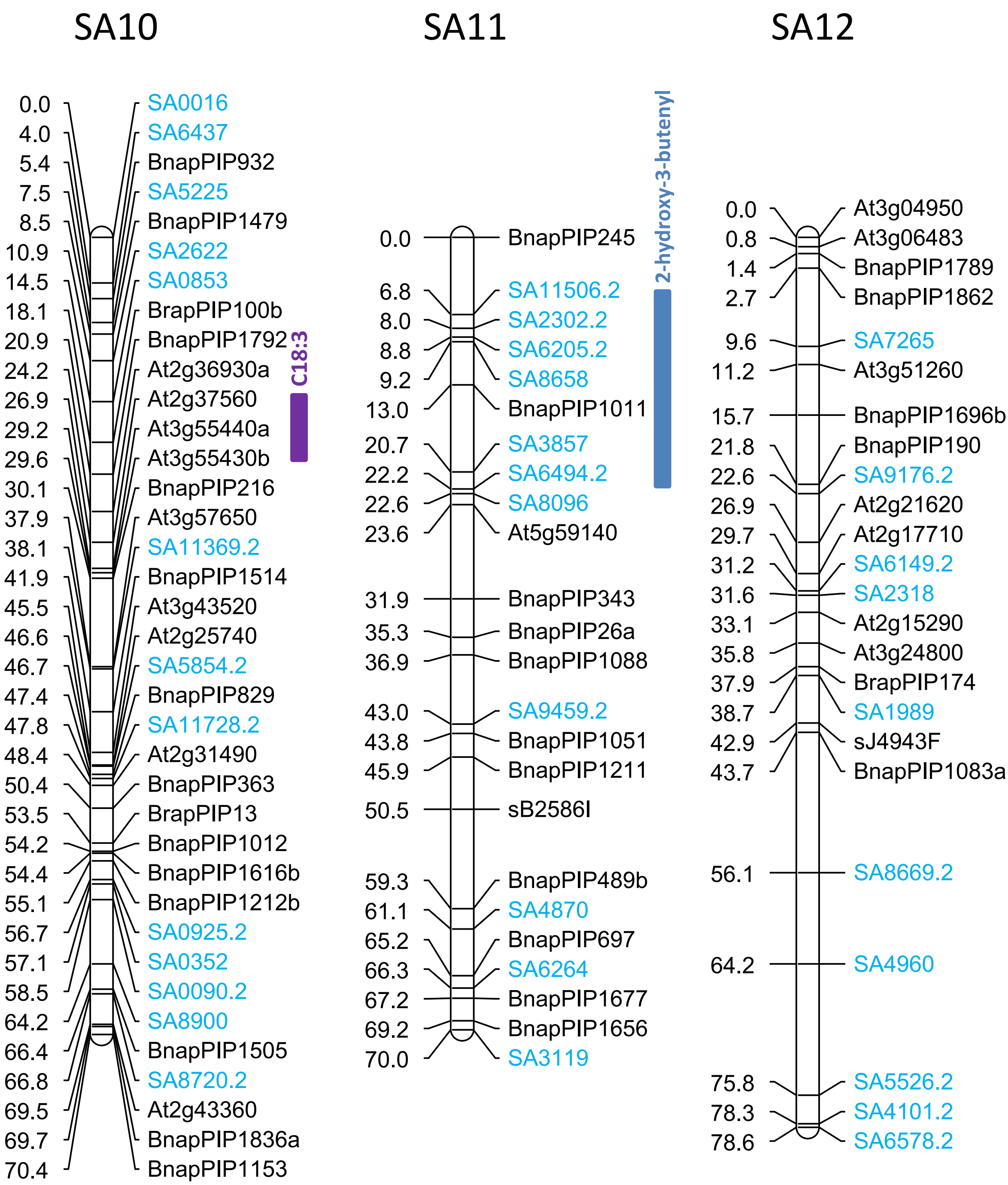

Figure S2: Sinapis alba genetic map and QTLs associated with fatty acid and glucosinolate content as per Javidfar and Cheng (2013) and Zeng et al. (2017). Purple (fatty acid) and blue bars (glucosinolate: Table S4) represent the location of QTLs associated with the listed traits. Markers in blue font represent additional KASP markers added via a GBS analysis. Markers in bold green font represent the markers present in the specific introgression line listed above the chromosome. Markers in orange amplify in *B. napus* and thus cannot be used to confirm the presence of the alba insert.
