# Supplementary material for: Introgressed Brassica napus × Sinapis alba enhance resistance to the crucifer flea beetle (Phyllotreta cruciferae)
Source: Pest Manag Sci. 2026 Jul 14;82(11):10752–64. doi: 10.1002/ps.71097 (PMC13665644; doi:10.1002/ps.71097)
Supplement: Supplementary file 2 [file PS-82-10752-s002.docx]

**Supplementary Information**

**Introgressed *Brassica napus* × *Sinapis alba* enhance resistance to the crucifer flea beetle (*Phyllotreta cruciferae*)**

**Running title:**  *P. cruciferae* resistance in introgressed lines.

Aziz Ullah^1^, Altaf Hussain^1^, Vicky Roslinsky^2^, Sally L. Vail^2^, Boyd A. Mori^1^

^1^Department of Agricultural, Food & Nutritional Science, University of Alberta, Edmonton AB

^2^Saskatoon Research and Development Centre, Agriculture and Agri-Food Canada, Saskatoon, SK

**Figure S1:** Crossing schemes for the two intergeneric pedigrees. Lines highlighted in green are *B. napus*, those in purple are *S. alba* and yellow highlighted lines are the selected *B. napus* lines with stable *S. alba* inserts. Generations highlighted in orange represent the stages that were screened to isolate lines with detected inserts. The red box displays the portion of the pedigree required to stabilize the genome and to consistently maintain the *S. alba* insert.

**A**

**B**

**E**

**D**

**C**

**Figure S3.** *Sinapis alba* line Emergo showing trichome density on **(A)** abaxial and **(B)** adaxial surfaces. The small red arrow in (**A**) and (**B**) indicates the trichome. Color images showing trichome development on the (C) seedling, (D) abaxial cotyledon, and (E) hypocotyls.

**B**

**A**

**Figure S4.** Variation in glucosinolate chemical diversity (Shannon index) among Brassicaceae lines **(A)** and its association with Phyllotreta cruciferae feeding damage **(B).**

**Figure S5.** Variable importance from random forest analysis of glucosinolate profiles from 6 selected Brassicaceae lines using *P. cruciferae* feeding damage ratings.

**Table S1:** ILP Primers utilized for selection during back cross generations

| **Developed Line** |  | ***S. alba* donor** | **Back-cross *B. napus* parent** | **Description**  (Fig. S1) | **Primer #1** | **Primer #2** |
| --- | --- | --- | --- | --- | --- | --- |
| BH14-21772 |  | SAB | 46A65 | *S. alba* SA03 insert*,* BC2 | BnapPIP890 | At4g33100 |
| VR19-176 |  | SAB | N99-508 | *S. alba* SA05 insert; BC4 | BnapPIP518 | At4g25140 |
| VR19-208 |  | SAB | N99-508 | *S. alba* SA07 insert; BC4 | BnapPIP1015 | BnapPIP1211 |
| VR19-289 |  | SAB | N99-508 | *S. alba* SA02 insert; BC4 | BnapPIP405 | BnapPIP1051 |
| VR19-133 |  | SAB | N99-508 | *S. alba* SA01insert; BC4 | BnapPIP558 | BnapPIP440 |
| VR19-156 |  | SAB | N99-508 | *S. alba* SA03 insert; BC4 | BnapPIP890 | At4g33100 |
| VR21-081 |  | Emergo | N99-508 | *S. alba* SA02 insert; BC3 | BnapPIP1051 | BnapPIP1536 |

| **Lines** | **Spring mean** | **Summer mean** | **W** | ***P*** |
| --- | --- | --- | --- | --- |
| Emergo | 41.5 | 0.0 | 55.0 | <0.05 |
| SAB | 43.0 | 30.0 | 31.5 | ns |
| AC-Excel | 35.5 | 28.5 | 25.0 | ns |
| N99-508 | 50.0 | 34.5 | 39.0 | ns |
| BH14-21772 | 18.0 | 27.0 | 21.5 | ns |
| VR19-289 | 12.0 | 23.0 | 7.0 | ns |
| VR19-208 | 22.5 | 64.5 | 3.0 | <0.05 |
| VR19-156 | 26.5 | 27.5 | 18.5 | ns |
| VR19-176 | 51.0 | 63.0 | 35.5 | ns |
| VR19-133 | 40.0 | 38.0 | 30.5 | ns |
| VR21-081 | 41.0 | 41.0 | 28.0 | ns |

**Table S2.** Comparison of flea beetle feeding damage between spring and summer experiments across 11 canola lines using Wilcoxon signed-rank tests. ns = not significant (P > 0.05)

**Table S3.** Pearson correlation degree of significance (*P*-value) for individual glucosinolates concentration (nmol/g DW) in different Brassicaceae lines used in the Experiment 2. Bolded *P*-values are significant at α=0.05

| **Glucosinolate** | **Group** | **Side-chain** |
| --- | --- | --- |
| Glucohirsutin | Aliphatic | 2-hydroxybut-3-enyl |
| 4-Hydroxyglucobrassicin | Indole | 4-hydroxy-3-indolmethyl |
| Glucocapparin | Aliphatic | 3-methylsulfinylpropyl |
| Glucoarabin | Aliphatic | 3-methylsulfinylpropyl |
| Glucoraphenin | Aliphatic | 4-methylsulfinylbut-3-enyl |
| Glucoalyssin | Aliphatic | 5-methylsulfinylpentyl |
| Epiprogoitrin | Aliphatic | 2-hydroxybut-3-enyl |
| Glucoerucin | Aliphatic | 4-methylthiobutyl |
| Glucoiberin | Aliphatic | 3-methylsulfinylpropyl |
| Progoitrin | Aliphatic | 2-hydroxy-3-butenyl |
| Sinalbin | Aromatic | 4-hydroxybenzoyl |
| Sinigrin | Aliphatic | 2-propenyl |
| Glucoraphanin | Aliphatic | 4-methylsulfinylbutyl |
| Glucotropaeolin | Aromatic | benzoyl |
| Gluconapin | Aliphatic | 3-butenyl |

**Table S4**: Classification and side-chain profiles of selected glucosinolates in different brassicaceae lines.
